# Spatial distribution and population dynamics of free-roaming (stray and semi-domiciled) dogs in a major Brazilian city

**DOI:** 10.3389/fvets.2024.1417458

**Published:** 2024-08-01

**Authors:** Carlos Eduardo de Santi, Wagner Antonio Chiba de Castro, Alessandra Cristiane Sibim, Renata Defante Lopes, Sandro Roberto Galvão, Giselli Maria Kurtz, Leandro Meneguelli Biondo, Louise Bach Kmetiuk, Alexander Welker Biondo

**Affiliations:** ^1^Zoonosis Control Center, City Secretary of Health, Foz do Iguaçu, PR, Brazil; ^2^Latin-American Institute of Life and Nature Sciences, Federal University for Latin American Integration (UNILA), Foz do Iguaçu, PR, Brazil; ^3^Latin-American Institute of Technology, Infrastructure and Territory, Federal University for Latin American Integration (UNILA), Foz do Iguaçu, PR, Brazil; ^4^Interdisciplinary Graduate Studies, University of British Columbia, Kelowna, BC, Canada; ^5^Zoonosis Surveillance Unit, City Secretary of Health, Curitiba, PR, Brazil; ^6^Department of Comparative Pathobiology, Purdue University, West Lafayette, IN, United States; ^7^Department of Veterinary Medicine, Federal University of Paraná (UFPR), Curitiba, PR, Brazil

**Keywords:** shelter medicine, dog population control, dog dynamics, animal welfare, zoonosis

## Abstract

**Introduction:**

Although estimate models have been proposed to determine free-roaming (both stray and semi-domiciled) dog populations, to date, no study has focused on the three major border areas of Brazil. Therefore, the present study assessed the free-roaming dog population of Foz do Iguaçu, a major far-west Brazilian city located in a three-border area (Brazil, Argentina, and Paraguay), which is considered among the top five Brazilian tourist destinations.

**Methods:**

Capture-release sampling was performed in three phases with a 6-month interval and 10-day duration of each phase, totaling 18 months, between 2018 and 2019.

**Results:**

A total of 1,273 dogs were estimated in the first [95% confidence interval (CI), 468–2,078 dogs], 904 in the second (95%CI, 452–1,355 dogs), and 1,564 in the third (95%CI, 521–2,607 dogs) capture phases in this area, suggesting a population density of 18.4 dogs/km^2^ (6.1–30.6 dogs/km^2^, 95% CI). Of all free-roaming dogs, 452/1,125 (40.2%) were stray with no confirmed ownership or household, whereas 672/1,125 (59.8%) had a known origin, among which 625/1,125 (55.6%) were semi-domiciled with ownership or a household, 36/1,125 (3.2%) were neighborhood dogs with maintainers, and 11/1,125 (1.0%) were owned by recycling material collectors and homeless individuals. The majority of the 1,125 dogs (862/1,125; 76.6%) had an ideal body condition score. The high outdoor access of owned dogs is likely caused by cultural behavior. However, because 533/1,125 (47.4%) of the free-roaming dogs presented with clinical abnormalities, irresponsible ownership may have negatively impacted dog health and welfare.

**Discussion:**

This study was the first to establish the density of free-roaming dogs, the ratio of stray and semi-domiciled dogs, and their dynamics over time in Foz do Iguaçu. The findings may serve as a warning for the high level of dog outdoor access and irresponsible guardianship, which may negatively affect animal health and welfare, leading to diseases, accidents, trauma, and animal cruelty.

## 1 Introduction

In pet management programs, free-roaming dogs are defined as dogs that do not have formal owners and households; the definitions for stray and semi-domiciled dog populations often overlap because both have free street access and are mostly indistinguishable (1). The distribution and dynamics of free-roaming dog populations may have considerable importance in planning and monitoring of population control strategies and animal welfare. These populations have been estimated using different methods (2), including mathematical modeling, to predict dog population dynamics (3).

In a theoretical scenario, the environmental carrying capacity has been considered the most effective factor impacting and modifying dog population dynamics (3). Previous studies on dog population distribution and dynamics have also aimed to achieve indicators and measurement methods, intended to serve as a basis to improve dog care and welfare, decrease dog population and density, stabilize population turnover, reduce public health risk, improve public perception and rehoming performance, and reduce the negative impact of dogs on wildlife and livestock (4).

Free-roaming dogs, identified as one of the primary issues in population management, result from neglect and abandonment, posing serious health, political, socioeconomic, and welfare threats (5). In addition, in several countries, the effectiveness of rabies vaccination programs depends on the control and management of the stray dog population (6). However, few studies worldwide have measured free-roaming dog populations. In Quito, Ecuador, three methodologies were used to assess free-roaming dogs: 5-km transect sampling, capture-recapture to estimate absolute population size, and calculating dog abundance in terms of the number of free-roaming dogs per kilometer (7). Free-roaming dog populations were estimated using the photographic sight–resight method in Herat, Afghanistan (8), and photographic and mark-recapture data in the Northern Mariana Islands, Spain (9). In Costa Rica, free-roaming dogs were assessed by the proportion of dogs wearing collars, indicating ownership (10). In São Paulo, southeastern Brazil, demographic characterization of dogs and cats was proposed using complex sampling with random selection in each administrative district (11), This method was used to assess domiciled and semi-domiciled dog populations in Jataí city, central western Brazil (12). Another study monitored the size and spatial distribution of a stray dog population on the main campus of the University of São Paulo, Brazil, recording 32–56 stray dogs from 2010 to 2011 (13).

The spatial distribution and population dynamics of free-roaming dogs vary according to environmental, social, economic, and cultural factors (14). Border areas in developing countries have been associated with low human development indices, environmental concerns, and illegal practices, which may present vulnerabilities from a One Health perspective (15, 16). To date, few dog population estimates and study exist for major border areas, particularly in developing countries. Therefore, this study assessed the free-roaming dog population in Foz do Iguaçu, Brazil, a major southern city located along the borders of Argentina and Paraguay.

## 2 Materials and methods

### 2.1 Ethics

This study was approved by the Committee on Ethics in the Use of Animals (protocol number 30/2018) of the University Center of Falls Dynamics, Foz do Iguaçu, Brazil. The study was approved by the City Attorney Office, State Attorney General Office, and State Council of Veterinary Medicine in Parana State. This study was also incorporated as an official activity for the Zoonosis Control Unit, Foz do Iguaçu Secretary of Health, and included the design, planning, training, and on-field capture and release of dogs. Thus, the use of financial and human resources was additionally approved by the City Health Commission and the Chamber of City Councilmen and issued by City Hall.

### 2.2 Study area

Foz do Iguaçu (25°32′49″S 54°35′11″W) is located on the far-west side of Paraná State, covering an area of 609.19 km^2^ and bordered by Brazil, Argentina, and Paraguay. It is one of the top five tourist destinations in Brazil. Among the 5,568 Brazilian municipalities, Foz do Iguaçu ranks 97th in population with 258,532 inhabitants (top 1.7%), 526th in the Human Development Index (HDI) with a high score of 0.751 (top 9.4%), and 59th in Gross Domestic Product (top 1.1%). The region has a subtropical climate, receives rainfall throughout the year, has an average temperature of 22.1^o^C, and is located within the Atlantic rainforest biome. This study was conducted in the urban area of the municipality, which covered 85.23 km^2^ and constituted ~99.17% of the population at the time of the study (17).

### 2.3 Dog capture release

**Figure 1:**
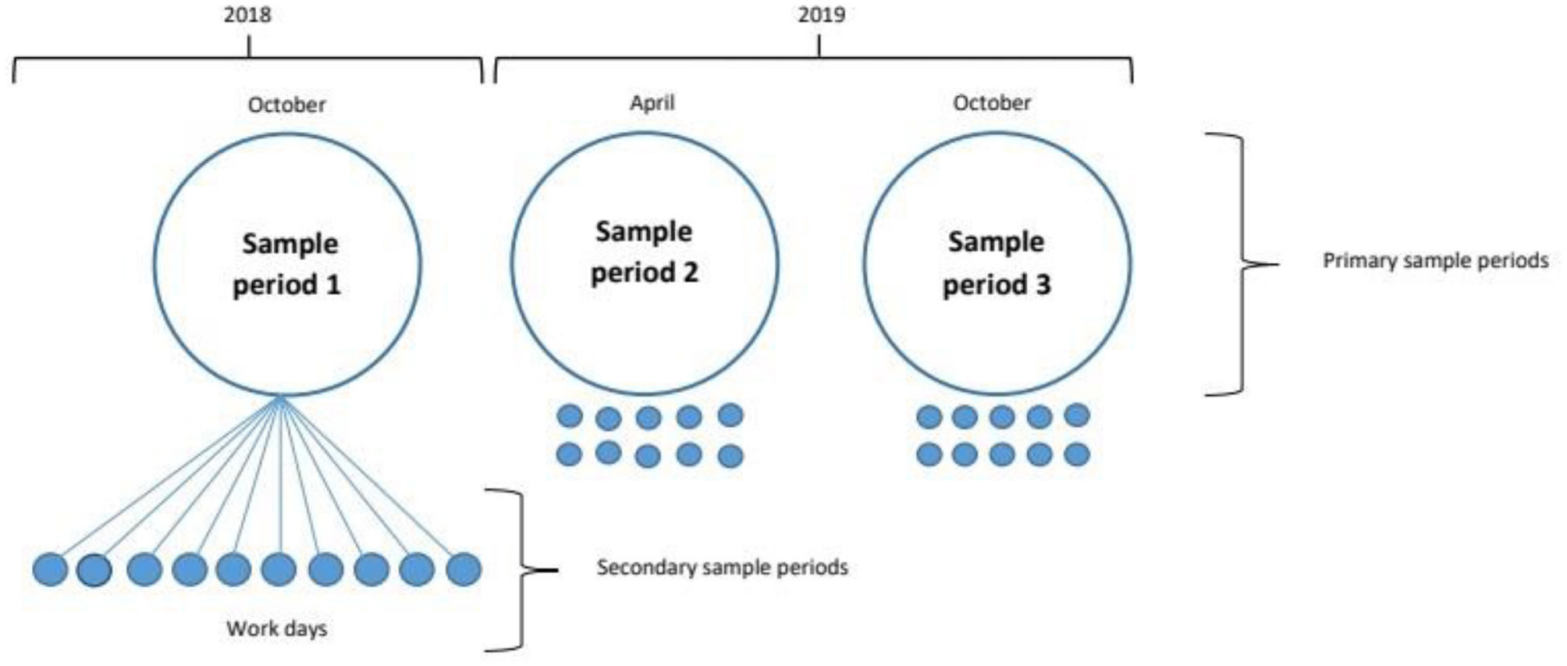
The free-roaming dog population study was conducted in three phases (primary sampling period), with an interval of 6 months between phases and 10 days of capture in each phase (secondary sampling period). Adapted from Smith et al. (18).

Capture-release population sampling was performed in three phases, separated by 6-month intervals, with each phase lasting 10 days, totaling 18 months. The first incursion occurred from 15 to 26 October 2018, the second from 15 to 26 April 2019, and the third from 14 to 25 October 2019 (Figure 1). No further samplings were possible due to the COVID-19 pandemic restrictions, which became officially effective in Brazil on 20 March 2020. The survey involved 37 professionals, including three veterinarians, seven technicians, and 27 inspectors, as well as eight veterinary medicine students. They were divided into five working groups, each comprising three professionals, one veterinary student, and one pickup truck. During captures, the coordinating veterinarian supervised the working groups and provided assistance by circulating on a motorcycle throughout the five city districts.

**Figure 2:**
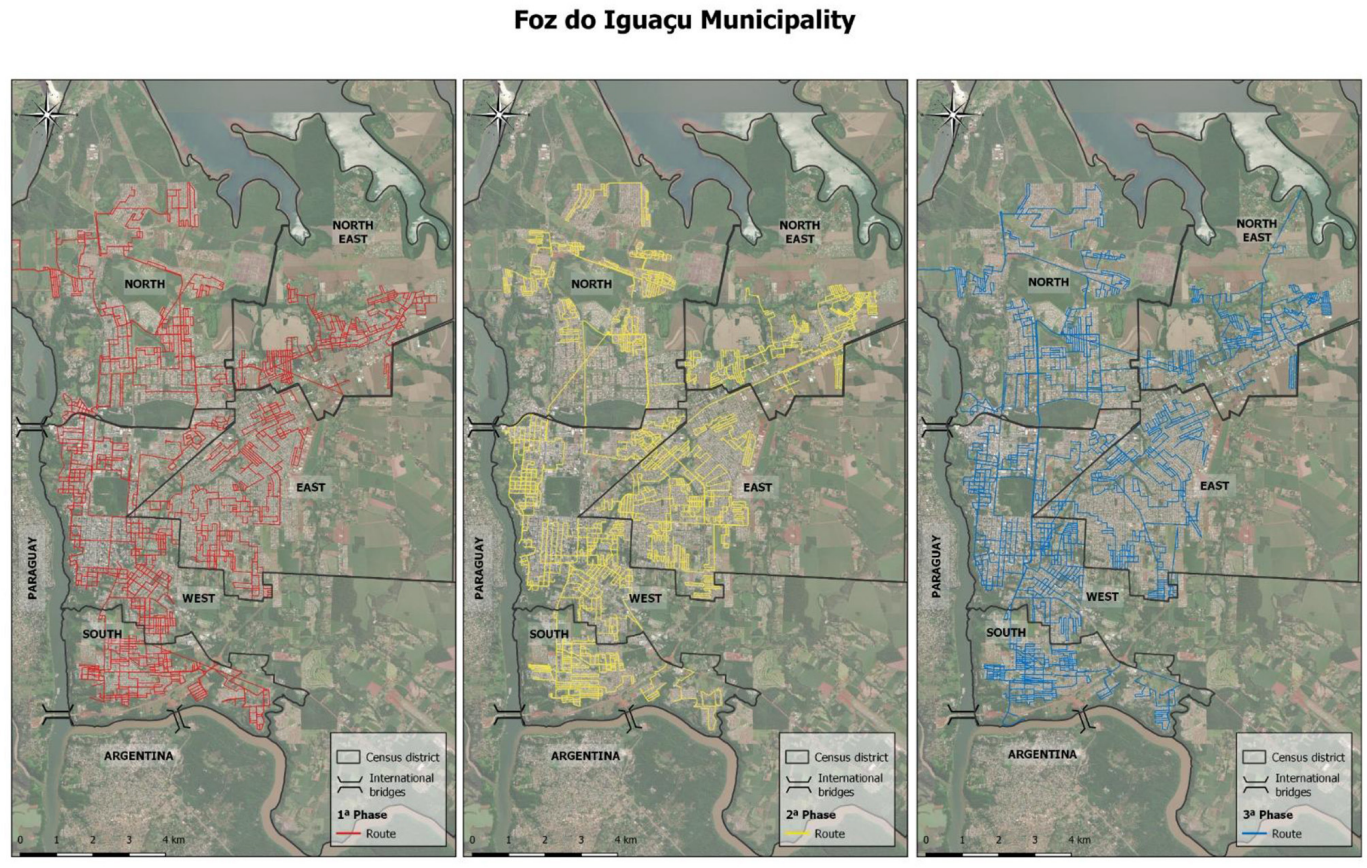
Urban area of Foz do Iguaçu, Brazil, showing the five censitary districts (in the north, south, east, west, and northeast regions), according to in-truck GPS monitoring, covered by the five groups over a 10-day period in each phase. Groups switched districts daily in a counterclockwise direction: from south to east, northeast, north, west, and back to south.

The urban area of Foz do Iguaçu was divided into five pre-existing districts, each with an allocated capture group to capture and recapture free-roaming dogs (Table 1). The groups randomly covered their district streets for ~3 min initially, capturing the first free-roaming dog they noticed. This procedure was repeated until eight dogs were captured (recaptured) within a 4-h daily period (Figure 2). Randomicity was ensured by interchanging the capture groups, who moved to the next contiguous district in a counterclockwise direction (from south to east, northeast, north, west, and back to south) each day. The captured dogs were registered, collars applied or checked, owners or households identified, and immediately released.

**Table 1:** Capture and recapture of dogs over an 18-month period in the urban area of the Foz do Iguaçu municipality, carried out between October 2018 and October 2019.

**Incursion**	**First**		**Second**		**Third**		**Total**	
**Date**	**October 2018**		**April 2019**		**October 2019**			
Total number of captured dogs	350	–	384	–	391	–	1,125	–
Total number of recaptured dogs (% rate)	10	2.9%	16	4.2%	9	2.3%	35	3.1%

Dogs were captured and restrained humanely to minimize animal handling stress, following the standard protocols of the Brazilian Federal Council of Veterinary Medicine. The dogs underwent a complete physical examination, where their estimated age, sex, breed, size, body score, and photographic records were obtained. A numbered collar was applied and anti-rabies vaccination and deworming were administered. Meanwhile, the working group searched the local neighborhood to determine whether the dogs had an owner or a maintainer. The dogs that had owners were returned to their households, following the guidance of guardianship and animal health, whereas the non-domiciled dogs were released at the same capturing site.

Dog ages were estimated based on teeth color, wear, tartar accumulation, and general physical aspects and were categorized into four age groups: puppies (up to 4 months old), young (from 4 months to 1 year), adults (from 1 to 7 years), and elderly (older than 7 years). Dog sizes were categorized as small, medium, and large. Body condition scores were based on a scale from one to nine, as previously established (19), and were categorized as under-ideal, ideal, and over-ideal. General examinations considered visible clinical signs such as dermatological problems, traumatic lesions, and exudative lesions, confirmed by a certified veterinarian.

### 2.4 Statistical analyses

To estimate the number of stray dogs in each of the three data collection phases of the study, the capture-recapture methodology premises were established. Reliable estimates of closed populations were based on the following premises: (1) population is steady during the period, (2) dogs maintain collars between captures/recaptures, (3) collars are properly recognized, (4) each dog has an even and constant likelihood of capture/recapture, and (5) dog collars do not alter the likelihood of capture or recapture.

The total number of free-roaming dogs in each phase was measured using the modified recapture method (20), based on the Laplace logic of dilution, which is the most indicated estimation method for free-ranging dogs (20).


$$\begin{array}{l} K=\;\overset{n}{\underset{1}{\sum }}\frac{T_{i}P_{i}}{R_{i}+1} \end{array}$$

where *K* denotes the estimated dog population, *T*_i_ represents the total number of dogs found in a capturing phase, *R*_i_ indicates the number of dogs found in a capture that were already caught (recaptures), *P*_i_ = *T*_i_ – *R*_i_ indicates the number of dogs captured for the first time, and n is the number of captures in a phase of the study.

To estimate the lower and upper limits of the 95% confidence interval (CI) for the free-roaming dog population (*K*), the Overton formula was applied (20) as below:


$$\begin{array}{l} K_{l},K_{u}=\left(1\pm \frac{2}{\sqrt{R_{i}}}\right) \end{array}$$

where *K*_l_ and *K*_u_ are the lower and upper limits of *K*.

## 3 Results

A total of 1,125 free-roaming dogs were captured; 350 in the first, 384 in the second, and 391 in the third phases, and the corresponding recurrence rates were 10 (2.86%), 16 (4.17%), and nine (2.30%) dogs (average = 3.11%).

The estimated numbers and population density of free-roaming dogs were 1,273 (468–2,078, 95% CI) and 14.9 dogs/km^2^ (5.5%−24.4%, 95% CI) in the first, 904 (452–1,355) and 10.6 dogs/km^2^ (5.3–15.9) in the second, and 1,564 (521–2,607) and 18.4 dogs/km^2^ (6.1–30.6) in the third incursions.

**Figure 3:**
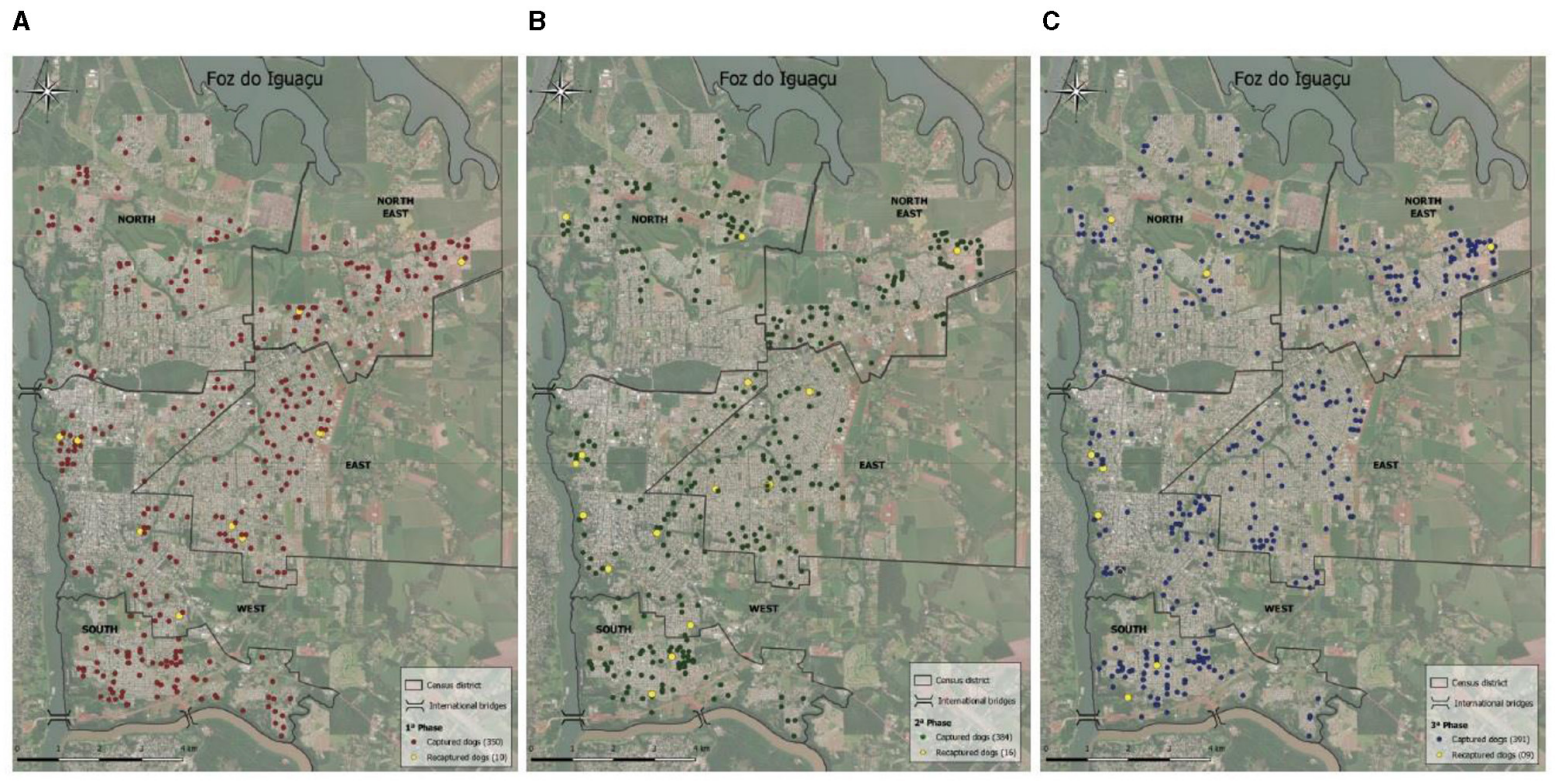
**(A–C)** Dispersion of captured dogs in the five districts of Foz do Iguaçu in each 6-month interval incursion.

**Figure 4:**
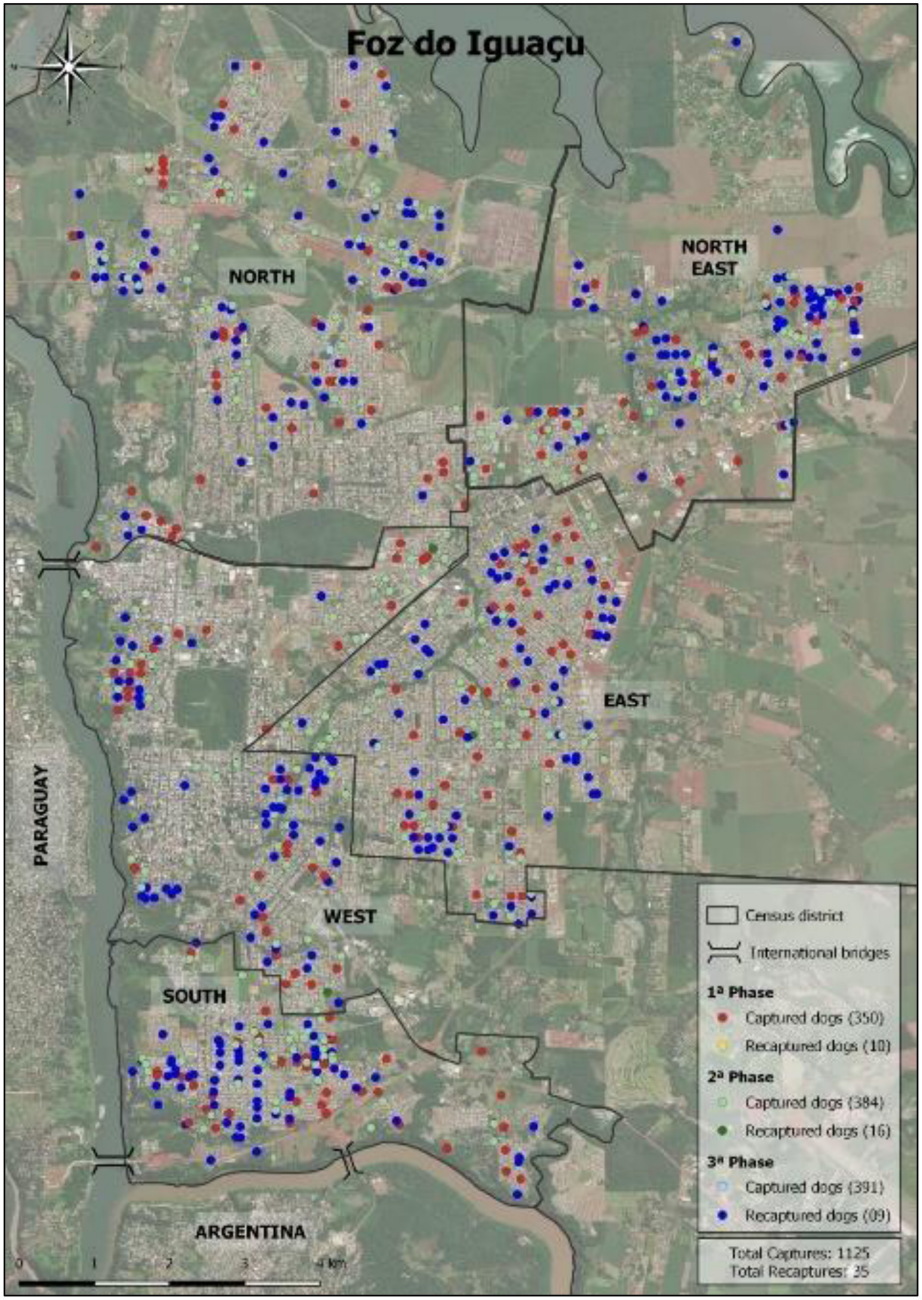
Overall spatial distribution of captured and recaptured free-roaming dogs from the three 6-month interval incursions in the five censitary districts of Foz do Iguaçu.

**Figure 5:**
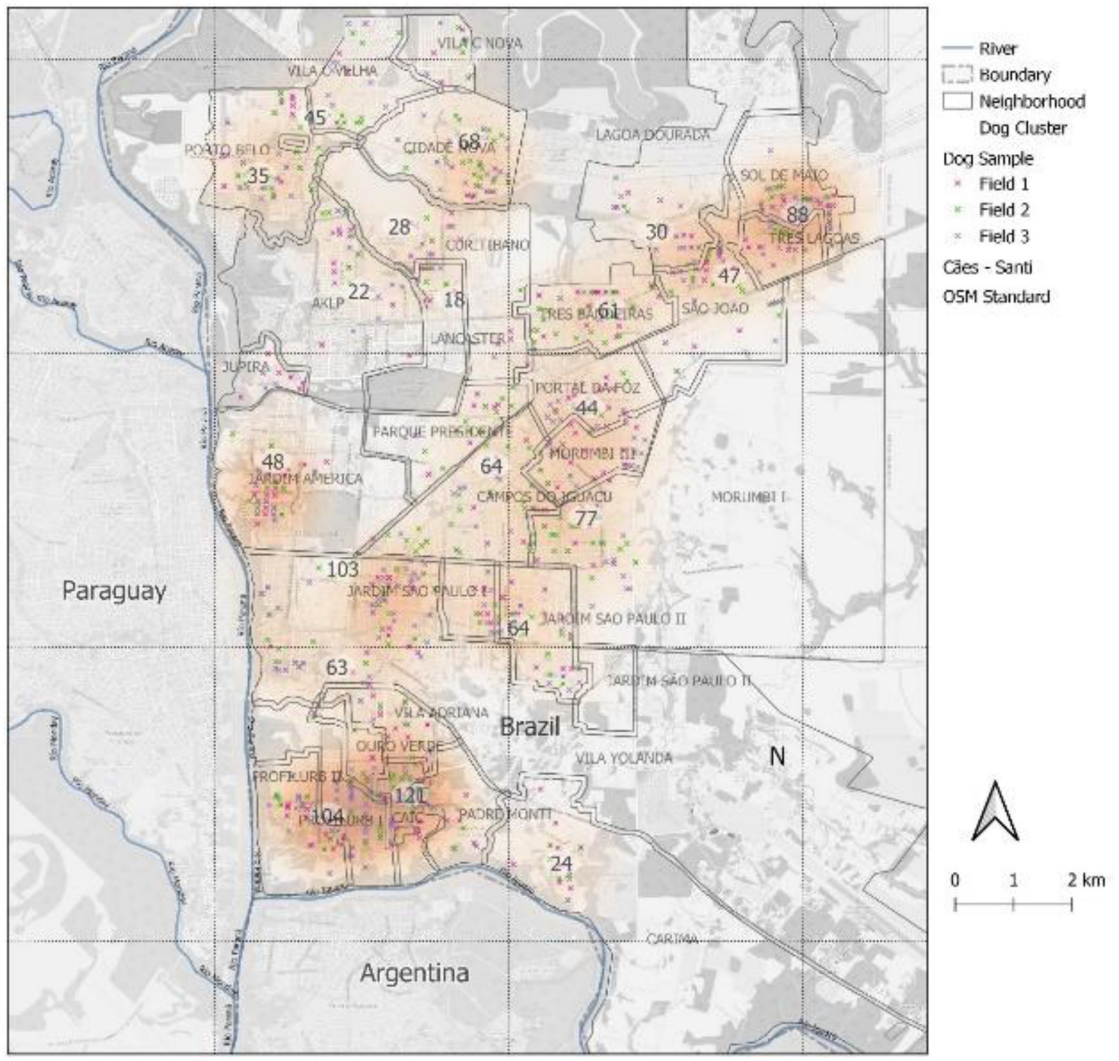
Spatial distribution and population density of free-roaming dogs of the urban area of Foz do Iguaçu city in the three study incursions conducted between October 2018 and October 2019.

The graphs of the distribution of captured dogs in the districts for each 6-month interval incursion show a pattern of distribution despite random capturing (Figures 3A–C). Figure 4 presents the plots of the overall spatial distribution of captured and recaptured free-roaming dogs during the three incursions in the five censitary districts. The neighborhood of America had one of the highest clusters of free-roaming dogs (Figure 5).

Overall, the study found 452/1,125 (40.2%) free-roaming dogs with no confirmed ownership or household, 672/1,125 (59.8%) dogs of known origin, and one dog with no definitive registration status. Of the dogs with known origin, 625/1,125 (55.6%) were semi-domiciled dogs with ownership or households, 36/1,125 (3.2%) were neighborhood dogs with maintainers, 8/1,125 (0.7%) were owned by recyclable material collectors, and 3/1,125 (0.3%) were claimed by homeless individuals. The body condition scores of the free-roaming dogs were mostly ideal, with 862/1,125 (76.6%) dogs, followed by 181/1,125 (16.1%) under-ideal dogs, 77/1,125 (6.84%) over-ideal dogs, and 5/1,125 (0.4%) dogs without a body condition score. The clinical examination results of the dogs revealed that 587/1,125 (52.2%) dogs were clinically healthy and 533/1,125 (47.4%) dogs had clinical abnormalities; 5/1,125 (0.4%) dogs did not undergo a clinical evaluation (Table 2).

**Table 2:** Body condition score, clinical examination, and origin of free-roaming dogs captured in the three incursions.

**Incursion**	**Total**	**Body condition score**			**Clinical examination**		**Origin**			
		**Under**	**Ideal**	**Over**	**Abnormal**	**Healthy**	**Stray**	**Semi-domiciled**	**Neighborhood**	**Other**
1^st^	350	50	283	16	159	191	116	211	19	3
2^nd^	384	68	287	25	192	192	203	166	10	5
3^rd^	391	63	292	36	182	204	133	248	7	3
**Missing**										
Total	1,125	181 (16.1)	862 (77.0)	77 (6.9)	533 (47.6)	587 (52.4)	452 (40.2)	625 (55.6)	36 (3.2)	11 (1.0)
Total	1,125	1,120			1,120		1,124			

Body condition was assessed as previously established (21).

Among the captured free-roaming dogs, 667/1,125 (59.3%) were male dogs, 453/1,125 (40.3%) were female dogs, and 5/1,125 (0.4%) dogs had no registered sex. A total of 767/1,125 (68.2%) were adult dogs (aged 1–7 years), 219/1,125 (19.5%) were young dogs (aged 4 months to 1 year), 93/1,125 (8.3%) elderly dogs (over 7 years old), 44/1,125 (3.9%) were puppies (up to 4 months old), and 2/1,125 (0.2%) dogs had no registered age. Overall, 1,064/1,125 (94.6%) were mixed, and 61/1,125 (5.4%) were breed dogs. Regarding dog size, 502/1,125 (44.6%) were medium, 468/1,125 (41.6%) were small, 147/1,125 (13.1%) were large, and 6/1,125 (0.5%) did not have a registered size. All collected data are presented in Table 3.

**Table 3:** Sex, age, breed, and size of free-roaming dogs captured in the three incursions.

**Incursion**	**Total**	**Sex**		**Age group**				**Breed**		**Size**		
		**Male (%)**	**Female (%)**	**Puppy (%)**	**Young (%)**	**Adult (%)**	**Elderly (%)**	**Mixed (%)**	**Breed (%)**	**Small (%)**	**Medium (%)**	**Large (%)**
1^st^	350	206	144	14	58	261	16	336	14	148	158	44
2^nd^	384	232	148	18	91	246	28	361	23	190	137	49
3^rd^	391	229	161	12	70	260	49	3,067	24	130	207	54
Total	1.125	667 (59.5)	453 (40.5)	44 (3.9)	219 (19.5)	767 (68.3)	93 (8.3)	1,064 (94.6)	61 (5.4)	468 (41.9)	502 (44.9)	147 (13.2)
Total considered	1.125	1.120		1.123				1.125		1.117		

Age was estimated based on visual observation of the coat and teeth, and based on age, the dogs were categorized as puppies (<4 months), young (4 months to 1 year), adult (>1 year), and elderly (>7 years). The percentages of[[Inline Image]] sex, age, breed, and size were calculated based on the total number of dogs with recorded data (total considered); the number of dogs that did not have data recorded were discarded.

## 4 Discussion

To the best of our knowledge, this is the first study to assess free-roaming dogs in a major border area of a developing country. Understanding the free-roaming dog population is crucial for public health and zoonosis control and prevention as well as for developing public policies related to animal health, welfare, and responsible ownership (14).

The estimated free-roaming dog population in Foz do Iguaçu at the end of the study was 1,564 (521–2,607, 95% CI), with a population density of 18.4 dogs/km^2^ (6.1–30.60 dogs/km^2^, 95% CI), resulting in a free-roaming dog to human ratio of 1:165. This ratio is based on a city population of 258,532, with 99.17% living in the urban area (22). The dog-to-human ratio varies from 1:1.1 in the Philippines (23) to 1:828 in Dhaka, Bangladesh (24). Although the ratio in this study is similar to those in Ilorin, Nigeria (1:139) (25) and rural areas of Bangladesh (1:120) (26), it was higher than the ratios in the urban areas of Herat, Afghanistan (1:315) (8) and Punjab, Pakistan (1:493) (27) and lower than those in Tanzania (1:14) (28); Chaudhari, India (1:14.3) (29); Sri Lanka (1:6.7) (30); Katmandu, Nepal (1:4.7) (31); and southern Mexico City (1:2.3) (32). An older World Health Organization report suggested a dog to human ratio of 1:8 for emerging countries, considering stray and domiciled dog populations (33). A 2023 survey in Curitiba City, the capital of Parana State (same as Foz do Iguaçu), with a population of 1,773,733, estimated a total of 584,661 dogs within the city, yielding a dog-to-human ratio of 1:3 (34), with 76,000 (13.0%) free-roaming dogs, including 59,000 (77.6%) semi-domiciled dogs and 17,000 (22.4%) stray dogs. Thus, as previously suggested, the impact of the dog-to-human ratio may be difficult to compare due to social, economic, demographic, environmental, and cultural factors (2).

In the study, the America neighborhood had one of the highest clusters of free-roaming dogs. Located in the western district near the Paraguayan border, it is characterized by high human traffic (83,000 people crossing the bridge per day on average), low income, slums, open-air dumps, and all types of waste. Another high free-roaming dog density area was the Três Lagoas neighborhood in the northeastern district, a densely populated, low-income area with open-air dumps, located farthermost from downtown. Similarly, the Porto Meira neighborhood in the southern district has an area of a 40-hectare slum with ~10,000 inhabitants, open-air dumps, and poor living and sanitary infrastructure, as well as a high population of free-roaming dogs.

Although unlikely, transit and migration of stray dogs may occur across Brazil through the Paraguayan and Argentinian bridge borders. Tens of thousands of workers and tourists cross the regional bridge (552 m long, 1,810 feet) between highly commercial urban areas of Brazil and Paraguay daily (the nearest bridge in the north ~220 km, 137 miles; and Argentina in the south) (Figure 1). On the other hand, the bridge between Brazil and Argentina (489 m long, 1,604 feet) is rarely crossed by foot (with most crossings by car), and it is farther from urban areas. The bordering Paraguayan city of Ciudad Del Este had an estimated population of 301,000, the bordering Argentinian city of Porto Iguazu had ~105,000 habitants, and Foz do Iguaçu had ~258,532 residents. Importantly, aside from international trade, the Iguaçu falls (between Brazil and Argentina) attract ~3.5 million visitors annually. Although there are no surveys to date on such international border crossings, researchers have estimated a total daily crossing (back and forth) of six and less than one stray dog through the bridges with Paraguay and Argentina, respectively.

Although the presence of stray dogs and feral cats can cause diseases, increasing cases of bacterial intestinal infections, mycosis, and toxoplasmosis in children and adults, as well as increase the number of bites by stray animals, no direct association could be established in this study. However, stray dogs can cause car accidents, cause aggressive encounters, serve as vectors for transmissible diseases, and result in environmental pollution by tearing open trash bags and defecating in public areas (5).

Despite the mandatory nationwide reporting of dog and cat aggression in Brazil due to rabies risk, no information on animal origin is included in online reports. Dog bites in Brazil are more likely to occur in lower-income neighborhoods, with an annual average incidence of 4.17 per 1,000 habitants in Curitiba (35). This study also showed that most dog bites, 35,755 out of 45,392 (78.8%) aggressive behaviors, were caused by dogs known to the victims (non-stray dogs), likely semi-domiciled dogs defending their territory (35). According to the Zoonosis Control Center, a total of 4,499 dog aggressions were reported in Foz do Iguaçu between 2019 and 2024, with no information on whether the dogs were owned or stray, including 900 cases in 2019, 705 in 2020, 814 in 2021, 865 in 2022, 859 in 2023, and 356 cases until May 2024.

This study on stray dogs highlighted the critical situation of border cities, as sterilization programs may not be a viable and effective strategy in regions with limited resources and high rates of animal abandonment (2). In addition, the high demographic turnover rates of dogs observed in such regions result in younger free-roaming populations, which are less likely to be vaccinated and more susceptible to diseases, particularly rabies and leishmaniasis, thereby posing a threat to public health (2).

Although a federal Department of Animal Rights and Protection was recently established by the Brazilian Government in 2023 (36), attention and resources have been mostly directed to major cities, located along the Atlantic Ocean shore, far from Brazil's inland borders. However, in highly populated urban areas such as Curitiba, the eighth largest Brazilian city and the state capital of Paraná (where Foz do Iguaçu is located), pet population control programs face difficulties; neutering or spaying and adoption have been identified to be the most effective control measures for stray dogs (1). However, different dog population management methods lack robust indicators and impacts of intervention, according to a recent systematic study worldwide (37). This study recommended improving reporting quality, study design, and modeling approaches in clearly defined target populations to allow determining causality, providing reliable cross-study data for a stronger evidence base to support interventions (37). Moreover, Brazilian studies have emphasized the importance of implementing educational programs to promote responsible dog guardianship and effective strategies against abandonment (2).

Although stray dog distribution may depend on the type of urban environment, they are mostly driven by food and water resources. This results in a balance between animals and carrying capacity, considered the most effective way to modify dog population dynamics (3). In this study, carrying capacity, including food and water supply, is provided throughout the city, as trash and leftovers can be found on streets, particularly over the weekends and holidays due to intense tourism and international trade activities in this three-border country area.

A significant finding was that 55.6% of the captured free-roaming dogs were semi-domiciled dogs, with owners allowing free outdoor access, which reflected animal neglect and was associated with 47.4% of clinical abnormalities and animal cruelty, resulting from the cultural behavior of irresponsible guardianship. This proportion is lower than the 77.6% of semi-domiciled dogs in Curitiba (34) but higher than 35.8% in São Paulo, the largest city in Brazil and South America (38). This study used identification collars to detect restricted or domiciled dogs with outdoor access; 7.8% of such dogs were found on the streets. These findings in these Brazilian studies highlight the need for educational actions to promote responsible pet guardianship, particularly focusing on changing cultural aspects and awareness of the risks of free-roaming dogs on streets, including zoonotic disease risk to public health, risks of trauma caused by accidents, such as cars hitting dogs, and environmental hazards due to feces and carcasses, summarized as One Health risks (11–13).

The remaining percentage of free-roaming dogs was considered unhealthy, given that 40.2% of stray dogs in Foz do Iguaçu were neglected, with cruelty resulting from animal abandonment. The percentage of stray dogs was higher than the 22.4% observed in Curitiba (34), suggesting a higher abandonment rate in the far-west countryside compared to the state capital. This result suggests a pattern of lower abandonment in the Atlantic coastal region, where most Brazilian state capitals, including São Paulo and Rio de Janeiro, are located.

The study found a male (59.5%) to female (40.5%) ratio of 1.5:1, which is consistent with the results of studies in other regions and countries. The findings indicate a predominance of male stray dogs, as previously observed in Herat, Afghanistan (8, 14, 39), rural areas (26), Dhaka, Bangladesh (24, 40, 41), and southern Mexico City (32, 42). This result may be attributed to the preference for male dogs in these neighborhoods, such as for personal protection, the avoidance of pregnancies of female dogs, a higher likelihood of abandonment (43), and a higher mortality rate of female dogs (44) due to successive pregnancies and deliveries on the streets (2, 45).

In this study, the majority of the captured semi-domestic dogs were male dogs (364/618, 58.9%) rather than female dogs (254/618, 41.1%). Although no estimates of the sex of domiciled dog populations were available, a massive anti-rabies vaccination campaign in 2014 showed a different pattern: 24,877/47,331 (52.6%) female dogs and 22,454/47,331 (47.4%) male dogs. This finding suggests that although the sex ratio of the domiciled dog population may be more evenly distributed, male dogs are more likely to become semi-domiciled dogs because of their territorial nature, surveillance, search for other dogs, particularly female dogs, and lesser human care (no undesired offspring).

Furthermore, this study found that most free-roaming dogs were adults (68.3%), followed by young (19.5%), elderly (8.3%), and puppies (3.9%), which agrees with the findings of previous studies that assessed age groups. This finding suggests a lack of subpopulation movements (such as local properties to streets), abandonment of adult dogs (18), or insufficient care for abandoned offspring, leading to low survival rates of puppies due to accidents, diseases, or intentional poisoning (44, 46). A high proportion of intact dogs in their reproductive stage on streets may worsen the problem with continuous offspring over time (28, 40). In addition, most free-roaming dogs in this study were predominantly mixed-breed dogs (94.6%), similar to the findings of other studies (2, 32).

Most free-roaming dogs were small (41.9%) and medium (44.9%) size; a small percentage were large dogs (13.2%), which may be because of their low life expectancy, food intake demand, aggressiveness, and general public fearfulness. The body condition scores of free-roaming dogs were predominantly ideal (77.0%), corroborating previous findings of 60.1% in Afghanistan (8) and 68.0% in urban areas of India (44). This finding suggests that these dogs were likely fed by owners, supporting the idea of that the population size is regulated by human demand (14).

In general, stray dogs tend to be more active in the spring season, with increased rutting and childbirth compared to winter, autumn, and summer months. Although this study was conducted in a humid subtropical climate region with hot summers and mild winters, a slightly higher percentage of stray female dogs was observed in spring (October, 41.1% in 2018 and 41.3% in 2019) than in fall (April, 39.0% in 2019). In addition, the percentage of adult dogs was slightly higher in spring (October, 74.8 in 2018 and 66.5% in 2019) than in fall (April, 64.2%). More importantly, the percentage of semi-domiciled dogs was significantly higher in spring (October, 60.4% in 2018 and 63.4% in 2019) than in autumn (43.2%). Thus, stray and semi-domiciled dogs in this study showed greater street roaming activity during spring compared to that in fall, likely due to reproductive motivation.

Although the fatness and body condition of stray dogs can vary, no significant differences in body weight were observed between spring and fall. This finding indicates an uninterrupted food supply, as tourism, commerce, and international trade may provide carrying capacity throughout the year. However, a slightly higher percentage of dogs presenting clinical signs of disease was observed in autumn (April, 50.0% in 2019) than in spring (October, 45.4% in 2018 and 34.5% in 2019). Despite increased reproductive activity in spring, leading to more interactions, dominance fights, and disease transmission, the impact on dog health may become more clinically evident only in the fall.

While free-roaming dog populations may struggle to remain healthy over time without veterinary assistance (44, 47), over half (55.6%) of the dogs were semi-domiciled, receiving some food and healthcare from owners. Nevertheless, 47.6% of the free-roaming dogs exhibited clinical abnormalities, including lameness, weight loss, and dermatological problems. By contrast, studies in Afghanistan found that 82.0% of free-roaming dogs were mostly healthy (8), and in Taiwan, where free-roaming dogs were monitored over 3 years (only 5.1%−8.8% had lameness and 14.2%−18.1% had skin problems) (48). Life on the streets may expose dogs to several life-threatening risks, such as car accidents, human aggression, animal fights, accidents, and diseases, leading to a shortened life expectancy (14), likely <3 years (49, 50).

Stray dogs in Latin America are often the result of abandonment, relinquishment, and undesired offspring (5). Stray dogs' daily activities mainly involve survival activities, including finding food, water, and shelter. In addition, semi-domiciled dogs roam the streets due to owners' convenience of letting dogs outside to eliminate urine and feces and inappropriate fencing, evidencing a lack of responsible guardianship (5).

The dog capture-recapture–release technique applied in this study, normally used in closed populations, is a reliable methodology. Once the premises were met, such as the stability of the dog population during the study period, retained dog collars, and individual free-roaming dogs between captures and recaptures recognizable, dog collars, and uniform and constant likelihood of capture and recapture (20). To ensure unbiased results, this methodology requires short-interval assessments, no loss of animal markers, and a homogeneous capture probability (2).

Despite various efforts, the city of Foz do Iguaçu lacked an official strategic plan for controlling the stray dog population (neutering/spaying), promoting responsible guardianship, ensuring animal welfare, and preventing zoonoses. Similarly, no official program was in effect at the time in the bordering Paraguayan and Argentinian cities, reflecting in the presence of stray dogs throughout the three-border country area. In fact, the present survey was conducted to serve as a basis for establishing effective stray dog strategies in the region, which could be extrapolated to other urban areas in Latin America and developing countries worldwide. Information on the origin and population density of free-roaming dogs is crucial for implementing effective public policies. Potential management strategies for free-roaming dogs include trap-neuter return (51), capture and removal of stray dogs to animal shelters and adoption (48), mandatory household restraining, and neutering/spaying (52–56). In addition, effective control and prevention programs for free-roaming dogs should include educational actions for responsible guardianship, zoonosis prevention, police enforcement, criminal prosecution of abandonment, awareness and prevention of abandonment, continuous adoption programs, and effective public waste management and waste collection (2).

Health strategies for neglected tropical disease control in the Iguaçu three-border area have been similar to those in Brazil, Argentina, and Paraguay, with a lack of epidemiological data and rural assessments, as noted in a recent systematic review (16). This study on stray dog spatial distribution and dynamics may provide practical significance for effective estimation, control, and preventive measures. Addressing the stray dog situation with scientific support can not only improve animal health and welfare but also reflect the ethical standards of a better society. In addition, stray and semi-domiciled dog populations may perpetuate disease transmission (14), particularly as reservoirs for local endemic zoonoses such as visceral leishmaniasis and rabies. In such a predisposed scenario, despite the first reported autochthonous case of visceral canine leishmaniasis in 2012 (the first case among all states), Foz do Iguaçu city had a total of 4,426 canine and 24 human visceral leishmaniasis cases from 2012 to 2020 (57). This continuous increase of dog cases with *Leishmania infantum* infection, followed by sporadic human cases, indicates dog-to-human transmission by infected *Lutzomyia longipalpis* sandflies (57).

Furthermore, rabies has become an increasing concern in the Foz do Iguaçu region because of the exposure of non-vaccinated stray and semi-domiciled dogs and feral cats to infected bats and other wildlife animals; this situation has increased since the discontinuation of the annual door-to-door pet anti-rabies vaccination performed in the 2000's (57, 58). According to the local Zoonosis Control Center, a total of 169 confirmed bat rabies cases have been reported since 2018 in Foz do Iguaçu, with 30 in 2018, 19 in 2019, 16 in 2020, 39 in 2021, 25 in 2022, and 40 in 2023. The last confirmed rabies cases in Foz do Iguaçu were registered in 2005 (one dog), 2002 (four dogs), 2001 (six dogs), 1999 (two dogs), 1998 (12 dogs), 1997 (four dogs and two cats), 1994 (one dog), 1990 (one dog), and 1985 (nine dogs). In addition, in 2002, two individuals from Paraguay with suspected symptoms crossed the border seeking treatment and were attended at the Foz do Iguaçu City Hospital and confirmed to be imported human rabies cases. Thus, this study can serve as a warning for rabies in stray and semi-domiciled dogs, as irresponsible guardianship can lead to a lack of anti-rabies vaccination.

This study has several limitations. The survey was designed for a longer period and a larger number of incursions; however, due to the COVID-19 pandemic, the incursion scheduled for April 2020 was canceled and the study ended prematurely. Despite the careful and continuous search, some free-roaming dogs may have been considered stray dogs because their owners or households could not be located during the incursions. As the study was conducted in 2018–2019 and the Brazilian National Census was conducted in 2010 and 2023, the demographic data used were based on estimates made by the Brazilian Institute of Geography and Statistics (59). In addition, the study did not assess whether dogs were neutered or spayed, which may have affected population dynamics and characteristics such as sex, age group, and recapture rate. Further studies should consider household surveys in areas with high densities of free-roaming dogs to better identify and compare owned and semi-domiciled dog populations.

## Data availability statement

The original contributions presented in the study are included in the article/supplementary material, further inquiries can be directed to the corresponding author.

## Ethics statement

This study was approved by the Committee on Ethics in the Use of Animals (protocol number 30/2018) at the University Center of Falls Dynamics, Foz do Iguaçu, Brazil. The study was conducted in accordance with the local legislation and institutional requirements.

## Author contributions

CS: Conceptualization, Data curation, Formal analysis, Funding acquisition, Investigation, Methodology, Project administration, Resources, Software, Validation, Visualization, Writing – original draft, Writing – review & editing. WC: Conceptualization, Data curation, Formal analysis, Funding acquisition, Investigation, Methodology, Project administration, Resources, Software, Supervision, Validation, Visualization, Writing – original draft, Writing – review & editing. AS: Data curation, Investigation, Methodology, Software, Writing – original draft, Writing – review & editing. RL: Data curation, Investigation, Methodology, Writing – original draft, Writing – review & editing. SG: Data curation, Investigation, Methodology, Writing – original draft, Writing – review & editing. GK: Data curation, Investigation, Methodology, Writing – original draft, Writing – review & editing. LB: Methodology, Software, Writing – original draft, Writing – review & editing. LK: Investigation, Validation, Writing – original draft, Writing – review & editing. AB: Supervision, Writing – original draft, Writing – review & editing.
